# Neoantigen load as a predictor of relapse in early-stage NSCLC: features that agonise and antagonise prognosis

**DOI:** 10.1007/s00262-025-04131-y

**Published:** 2025-08-06

**Authors:** Linda Ye, Ian Dick, Bruce W. Robinson, Jenette Creaney, Alec Redwood

**Affiliations:** 1 National Centre for Asbestos Related Diseases, Medical School, University of Western Australia, Nedlands, Australia; 2 Department of Medical Oncology, Royal Perth Hospital, Perth, Australia; 3 School of Biomedical Science, University of Western Australia, Perth, Australia; 4 Institute for Respiratory Health, University of Western Australia, Perth, Australia; 5 Department of Respiratory Medicine, Sir Charles Gairdner Hospital, Perth, Australia

**Keywords:** Non-small cell lung cancer, Neoantigen, Prognosis, Biomarker

## Abstract

**Background:**

Neoantigen-specific immune responses may help prevent cancer recurrence. We evaluated whether neoantigen load and/or properties could predict survival in early-stage non-small cell lung cancer (NSCLC).

**Methods:**

Whole-exome sequencing (WES) data from 89 resected early-stage NSCLC patients were used to identify non-synonymous single-nucleotide variants (nsSNV) and to predict major histocompatibility complex class I neoantigens. Neoantigen load, differential aggretopicity index (DAI), neoantigen frequency (number of neoantigens per nsSNV) and neoantigen promiscuity (ability to bind multiple human leucocyte antigen (HLA) alleles) were assessed for association with time to recurrence (TTR) and recurrence-free survival (RFS).

**Results:**

Higher neoantigen load was independently associated with longer TTR (*p* = 0.028). A greater number of neoantigens with high DAI (≥ 10) were associated with improved TTR (*p* = 0.008) whilst increased neoantigen promiscuity correlated with both longer TTR (*p* = 0.007) and RFS (*p* = 0.010). Conversely, elevated neoantigen frequency predicted a worse prognosis (TTR *p* = 0.016).

**Conclusions:**

These data support a role for T cells in on-going immunosurveillance in resected NSCLC patients and suggest that both quality and quantity of neoantigens are important drivers of anti-cancer immunity and may inform future biomarker and immunotherapy development.

**Supplementary Information:**

The online version contains supplementary material available at 10.1007/s00262-025-04131-y.

## Introduction

Non-small cell lung cancer (NSCLC) is associated with significant mortality and morbidity. Although surgery can cure patients with localised disease, up to 55% suffer cancer recurrence within 5 years [1]. Traditional clinical factors, such as TNM staging, are imperfect predictors of outcome, highlighting the need for more precise biomarkers to stratify relapse risk.

Recent advances in the use of immune checkpoint inhibitor (ICI) in the perioperative setting have demonstrated a 28% improvement in overall survival [2], highlighting the importance of a protective anti-tumour immune response even in the early-stage setting. Neoantigens—novel peptides that arise from tumour-specific mutations—are the primary targets of T cells [3], and neoantigen-specific T cells responses have been associated with pathological response [4]. It is likely that these responses play a role in the systemic surveillance of micrometastasis after surgery. The extent of the neoantigen-specific immune response may protect from recurrence.

The previous studies in early-stage NSCLC have examined tumour mutational burden (TMB) as a surrogate for neoantigen load. Results from these studies have been contradictory, with some studies showing that a high TMB is associated with a favourable outcome [5], whilst others have reported that TMB is negatively prognostic [6], or has no association with patient survival [7, 8]. These inconsistent findings imply that TMB alone is insufficient for predicting prognosis in early-stage NSCLC.

Prior studies have also examined predicted peptide—major histocompatibility complex (MHC) binding affinity (IC50 ≤ 500 nM) as a prognostic biomarker in early-stage NSCLC with similarly inconsistent results. Gong and colleagues reported that higher neoantigen load was associated with improved survival in patients with early-stage squamous cell carcinoma (SCC) [8]. In contrast, McGranahan and colleagues found no association with prognosis in SCC patients, but demonstrated a positive association with overall survival in adenocarcinoma (ADC) [9]. These findings suggest that additional factors influencing neoantigen immunogenicity must be considered.

**Fig. 1:**
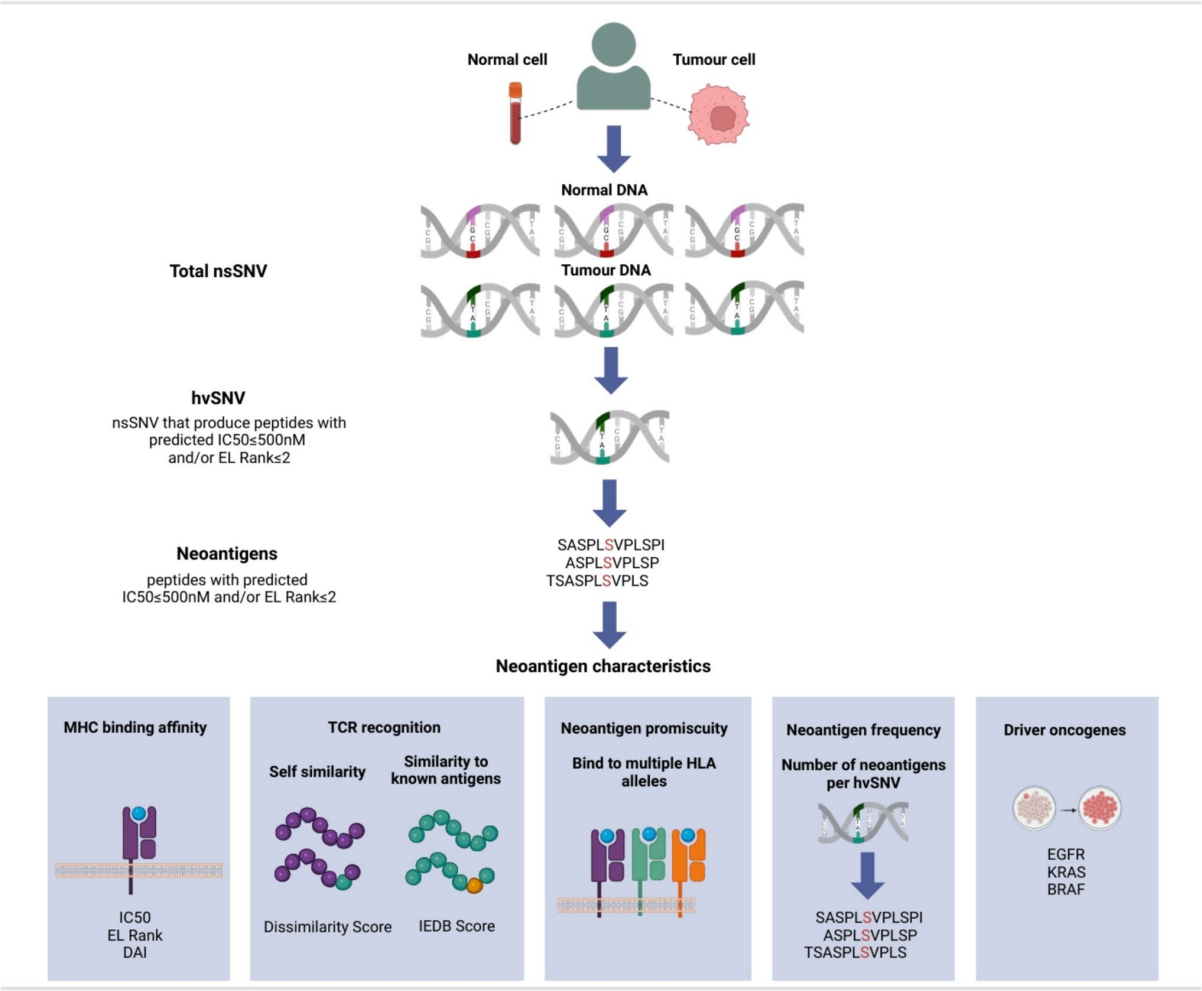
MHC class I neoantigen prediction and neoantigen characteristics that may influence immunogenicity. nsSNV, Non-synonymous single-nucleotide variant; hsSNV, High value non-synonymous single-nucleotide variant; EL rank, Eluted ligand % rank; DAI, Differential aggretopicity index; IEDB, Immune Epitope Database

Neoantigen immunogenicity can be further characterised using metrics beyond binding affinity alone. The Eluted Ligand (EL) rank score combines binding affinity training data with peptide elution data [10] and may better predict antigen presentation and processing. Other features of potential importance include the differential aggretopicity index (DAI), which compares the binding affinity of the mutated and wildtype peptides [11]. DAI reflects to a degree the dissimilarity between a mutated peptide and its non-mutated counterpart, and hence, neoantigens with a high DAI may be less subject to self-tolerance [12]. A DAI cutoff of greater than 10 has previously been shown to correlate with patient survival across tumour types, as well as intratumoural T-cell responses and improved rate of experimental neoantigen validation [12, 13]. The probability of T-cell receptor (TCR) recognition, estimated by comparison to known antigens (i.e. Immune Epitope Database (IEDB) score) [14, 15] or by assessing dissimilarity to native proteins (dissimilarity score) [15, 16], has also been associated with neoantigen immunogenicity. Neoantigen promiscuity, defined as the capacity of a neoantigen to bind more than one HLA allele, has shown to yield more experimentally validated neoantigens [17], possibly because binding to multiple alleles increases the chance for HLA presentation and makes the presentation of neoantigens more resistant to loss of specific alleles [18]. Lastly, the presence of oncogene-derived neoantigens, which suggests neoantigen clonality, is associated with upregulation of genes associated with effector T-cell activation, and high clonal neoantigen burden has been linked to improved cancer outcomes [19] (Fig. 1).

We hypothesise that the survival outcomes of patients with resected early-stage NSCLC are associated with the quantity and/or quality of tumour-derived neoantigens. In this study, we used an in silico neoantigen prediction pipeline on whole-exome sequencing (WES) data from a cohort of 89 patients with resected early-stage NSCLC from the University of Texas M.D. Anderson Cancer Centre (MDACC) to determine whether MHC class I (MHC-I) neoantigen load and specific properties defining neoantigen immunogenicity, are associated with longer time to recurrence (TTR) and recurrence-free survival (RFS).

## Materials and methods

### Patient dataset

We obtained permission to access WES, HLA typing and clinical data of patients with resected Stage I–III NSCLC from a published MDACC dataset [20]. Within this dataset, WES data of tumour and matched blood were available for 95 patients. Six cases had no somatic variants and were excluded, resulting in 89 patients being included in the final analysis. The survival endpoints examined were TTR defined as the time interval between date of surgical resection and date of cancer recurrence or last follow-up, and RFS defined as the time interval between date of surgical resection and date of cancer recurrence, death or last follow-up, whichever occurred earliest.

### Somatic variant calling

WES was downloaded as BAM files from MDACC EGAD00001005956 dataset [20]. The BAM files had been aligned to the GRCh37 (b37) reference and processed according to the GATK best practice guidelines [21]. We then used a combination of variant callers; SomaticSniper [22], Varscan2 [23] and Mutect2 [24], to identify somatic mutations. For each variant calling programme, a filtering procedure, using the default settings for each, was used to select high confidence variants. Vcfs from SomaticSniper and Varscan2 were combined using the vcf-isec function, a component of VCFtools. Output was then combined with that of Mutect2 and duplicates removed. The combined output of the three callers was analysed. The GRCh37 coordinates for these vcfs were lifted over to the corresponding GRCh38 coordinates using the Crossmap programme [25], and the resulting converted vcfs were then annotated using SnpEff version 5 [26].

### Neoantigen prediction and characterisation

For each nsSNV in the annotated vcf files, MHC-I neoantigen predictions were determined using antigen.garnish [15], which produced netMHCpan4.1 eluted ligand (EL) rank and binding affinity predictions, the DAI, dissimilarity score and IEDB score for mutant associated peptides of lengths 8–14. Neoantigens are defined here as unique peptides with IC50 ≤ 500 nM or EL rank ≤ 2. NSCLC associated oncogenes were identified using the COSMIC database to determine the number of neoantigens arising from oncogenic driver genes [27].

### TCR repertoire analysis

To characterise the TCR repertoire of the primary tumour, the publicly available TCR β-chain CDR3 region sequencing data from the MDACC dataset was examined (i.e. immuneACCESS platform). TCR diversity was quantified using the Inverse Simpson’s Diversity Index [28]. TCR clonality was quantified using the Simpson’s clonality index [29].

### Statistical analysis

Mann–Whitney U-test was used for two-group comparison, Kruskal–Wallis H test was used for multiple group comparison. Spearman’s rank non-parametric correlation analysis was applied to measure the correlation between two continuous variables. The Kaplan–Meier method and log-rank test was performed for associations with TTR and RFS using univariate and multivariate analyses. To generate the Kaplan–Meier survival curves, continuous variable was dichotomised according to an optimal cutoff value determined using Cutoff Finder [30]. Receiver operator characteristics (ROC) analysis was used to determine the predictive value of each variable for relapse-free probability. To determine the predictive value of combining multiple neoantigen properties for relapse-free probability, logistic regression analyses were performed on categorical variables and continuous variables after log transformation. The probability of an individual being relapse-free at 3 years was calculated from the logistic regression coefficients of the variables, both individually and in combination, using the logistic regression equation as previously described [31]. These probability estimates were then dichotomised into ‘high’ and ‘low’ groups for correlation with survival outcomes using the SPSS ROC procedure to perform AUC analysis, using the Youden index approach to predict optimal cutoff values.

Statistical analysis was performed using IBM SPSS statistics version 29.0.0.0 and GraphPad Prism 9 software. A p value of < 0.05 was considered statistically significant.

## Results

### Clinicopathological characteristics of MDACC dataset

A total of 89 patients from the MDACC dataset were included in our study and included 52 males (58%). Eighty-one patients (91%) were former or current smokers. Data on age and performance status were not available. Fifty-nine (66%) patients had adenocarcinomas (ADC) (including 1 adenosquamous), and 30 patients had SCC (34%). EGFR mutations were present in 9 patients (10%), and KRAS mutations were present in 19 patients (21%). As expected, all were found in ADCs. There were 45 patients with stage I (50%), 26 with stage II (29%) and 18 with stage III (20%) disease, all of whom underwent surgery with curative intent without prior therapy. Data on adjuvant and subsequent therapies were not available. The median follow-up was 5.4 years (5 months–12.8 years). Patient characteristics are listed in Table 1.

**Table 1:** Clinical and pathological characteristics of early-stage resected NSCLC cases (*n* = 89)

Characteristics		*N* (%)
Gender	Male	52 (58)
	Female	37 (42)
Stage	I	45 (51)
	II	26 (29)
	III	18 (20)
Histology	Adenocarcinoma	58 (65)
	Adenosquamous	1 (1)
	Squamous	30 (34)
Smoking	Yes	81 (91)
	No	8 (9)
EGFR	Mutant	9 (10)
	Wildtype	80 (90)
KRAS	Mutant	19 (21)
	Wildtype	70 (79)

### Neoantigen landscape of early-stage non-small cell lung cancer

To profile the neoantigen landscape of early-stage NSCLC, first we determined total nsSNV load, high value SNV (hvSNV) load and neoantigen load (Fig. 1). Neoantigens are defined here as unique peptides with IC50 ≤ 500 nM or EL rank ≤ 2. hsSNV is defined as a nsSNV giving rise to at least one such neoantigen.

**Fig. 2:**
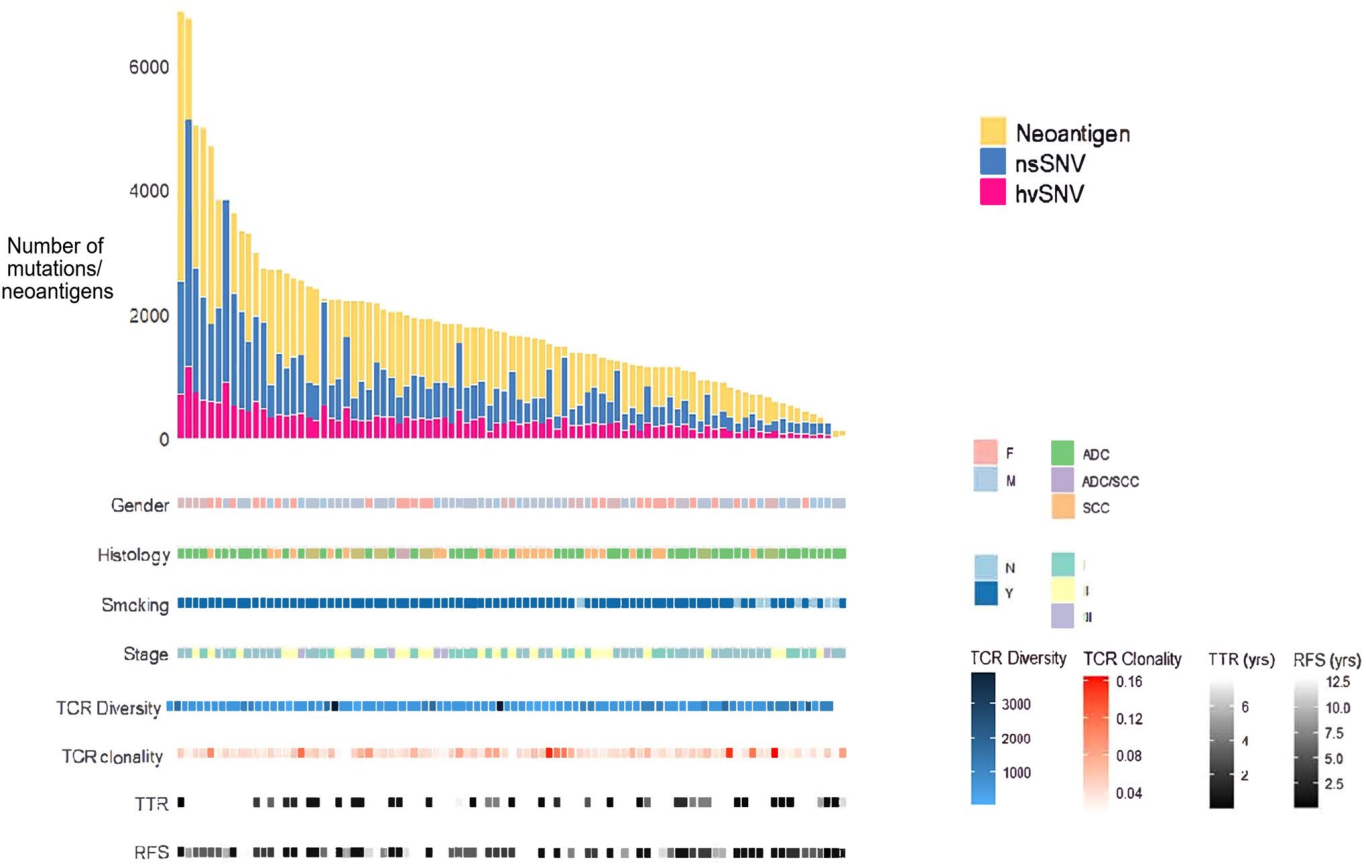
Mutational and MHC-I neoantigen profile, and clinical characteristics in 89 resected NSCLC patients. Clinical and pathological parameters for each patient shown: nsSNV load, hvSNV load, predicted MHC Class I neoantigen load, gender, TNM stage, smoking history, TCR diversity, TCR clonality, TTR and RFS. NSCLC, Non-small cell lung cancer; nsSNV, Non-synonymous single-nucleotide variant; hvSNV, High value non-synonymous single-nucleotide variant; MHC, Major histocompatibility complex; TCR, T-cell receptor; TTR, Time to recurrence; RFS, Recurrence-free survival

Across the 89 tumours, we identified 85,350 nsSNVs, of which 26,028 were classified as hvSNV. The median nsSNV load per tumour was 800 (25–5126) and the median hvSNV load was 262 (10–1163) (Fig. 2). In total, 165,682 predicted MHC-I neoantigens were identified, with a median of 1670 neoantigens per tumour (range 127–6882). The median TMB was 23.5 Mut/Mb, which is consistent with that reported for early-stage NSCLC [32]. As expected, nsSNV load and hvSNV load showed strong positive correlation (Spearman’s rho 0.976, *p* < 0.001).

Overall, neoantigen load strongly positively correlated with both total nsSNV load (Spearman’s rho 0.877, *p* < 0.001) and hsSNV load (Spearman’s rho 0.915, *p* < 0.001) (Supp Fig. 1). The majority of the predicted neoantigens had an EL rank ≤ 2 (73.5%), whilst the rest were selected due to IC50 ≤ 500 (47.1%). 20.7% of predicted neoantigens satisfied both EL rank ≤ 2 and IC50 ≤ 500. 19.4% of neoantigens per tumour were classified as strong binders with EL rank ≤ 0.5, and 6.8% had IC50 ≤ 50 (Supp Fig. 2A, B).

We next evaluated properties of neoantigens. Only a small proportion of neoantigens per patient (mean 1.6 ± 0.7%) had a DAI ≥ 10, a threshold previously linked to improved survival and T-cell responses [13] (Supp Fig. 2C).

Similarly, based on metrics proposed by Luksza and colleagues [14, 15], predicted TCR recognition probability was low, with 0.2 ± 0.2% of neoantigens having a dissimilarity score ≥ 0.75 and 1.9 ± 1.0% an IEDB score ≥ 0.9 (Supp Fig. . 2D, E).

Analysis of neoantigen promiscuity found 19.2 ± 9.8% of neoantigens could bind to more than one HLA allele (Supp Fig. 2F). In the cohort, 43 patients (48%) expressed 6 HLA alleles, 34 (38%) expressed 5 alleles, 10 (11%) expressed 4 alleles and 2 (2%) were homozygous, expressing only 3 alleles. Of the total 29,593 promiscuous neoantigens across the cohort, 54.8% were predicted to bind across different HLA alleles (i.e. both HLA-A and HLA-B) indicating true cross-HLA binding rather than redundancy within supertypes (Supp Fig. 3).

Finally, we found 44 oncogenes gave rise to neoantigens, representing 1.0% of all hvSNV and 0.9% of all predicted neoantigens (Supp Fig. 2G). Neoantigens were most commonly derived from CSMD3, TP53, KEAP1, KRAS, GRIN2A, BIRC6, PTPRT, NOTCH1, PTPRD and EPHA3 oncogenes (Supp Table 1).

When comparing the neoantigen characteristic across different clinical subgroups, there were no significant differences between the nsSNV load, hvSNV load, neoantigen load or neoantigen characteristics between ADC and SCC, and different tumour stages (Supp Table 2 and Supp Table 3). As expected, non-smokers and those with EGFR mutant cancers had significantly lower nsSNV, hvSNV and neoantigen load compared to smokers and EGFR wildtype cancer, respectively (Supp Table 4 and Supp Table 5). However, there were no differences in the neoantigen characteristics between the two groups. KRAS mutant tumours did not differ in their nsSNV, hvSNV or neoantigen load compared to KRAS wildtype counterparts. However, they did harbour a greater proportion of neoantigens with DAI ≥ 10 (Supp Table 6).

### Predicted nsSNV, hvSNV and neoantigen load all correlated with improved prognosis

**Fig. 3:**
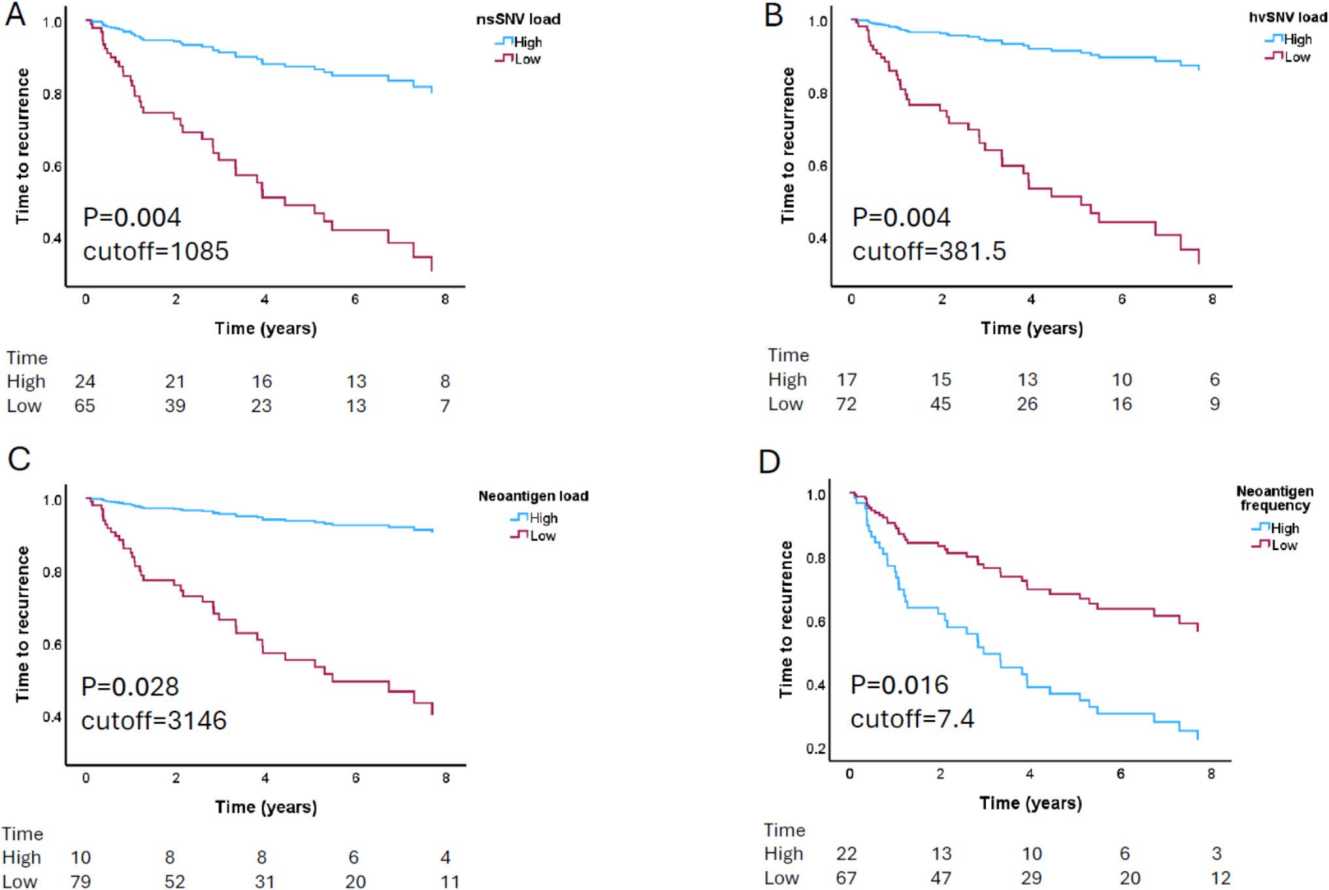
nsSNV, hvSNV and neoantigen load correlate with improved TTR, whilst neoantigen frequency correlate with shorter TTR in early-stage resected NSCLC (*n* = 89). Multivariate Cox regression analysis of TTR according to **a** nsSNV load, **b** hsSNV load, **c** neoantigen load and **d** neoantigen frequency. High and low groups are dichotomised by the optimal cutoff determined using Cutoff Finder. NSCLC, Non-small cell lung cancer; nsSNV, Non-synonymous single-nucleotide variant; hvSNV, High value non-synonymous single-nucleotide variant; TTR, Time to recurrence

In advanced cancers, TMB and neoantigen load have both been reported as positive prognostic biomarkers [9, 33, 34]. Their value in early-stage NSCLC has not been defined. We examined the correlation between nsSNV, hvSNV and neoantigen load with TTR and RFS. In multivariate Cox regression analysis, adjusting for sex, pathologic stage, smoking history, histological subtype, EGFR and KRAS mutation status, all three metrics were significantly associated with longer TTR (nsSNV *p* = 0.004, hvSNV *p* = 0.004 and neoantigen *p* = 0.028) (Table 2 and Fig. 3). There was a trend towards a positive association between nsSNV and hsSNV load and RFS (nsSNV *p* = 0.069 and hvSNV *p* = 0.064).

**Table 2:** Multivariate analysis of TTR and RFS predictors in early-stage resected NSCLC (*n* = 89)

Variables		Univariate						Multivariate^a^					
		TTR			RFS			TTR			RFS		
		HR	95% CI	*p*	HR	95% CI	*p*	HR	95% CI	*p*	HR	95% CI	*p*
Gender	F versus M	0.497	0.25–0.98	0.044	0.893	0.55–1.46	0.653						
Histology	SCC versus Adeno	0.683	0.33–1.40	0.299	0.695	0.40–1.20	0.191						
Stage	I												
	II	2.239	1.06–4.72	0.034	1.387	0.78–2.45	0.261						
	III	4.130	1.86–9.16	< 0.001	1.762	0.90–3.44	0.098						
Smoking	Never versus Ever	0.864	0.26–2.82	0.808	0.488	0.21–1.16	0.104						
EGFR	Mutant versus WT	0.460	0.11–1.91	0.285	1.250	0.56–2.77	0.583						
KRAS	Mutant versus WT	1.720	0.88–3.35	0.111	1.215	0.70–2.12	0.491						
nsSNV load	Per 100 unit increase	0.928	0.87–0.99	0.017	0.975	0.94–1.01	0.124	0.912	0.86–0.97	0.004	0.969	0.94–1.00	0.069
hvSNV load	Per 100 unit increase	0.773	0.62–0.96	0.018	0.886	0.77–1.02	0.090	0.720	0.58–0.90	0.004	0.874	0.76–1.01	0.064
Neoantigen load	Per 1000 unit increase	0.763	0.56–1.04	0.092	0.885	0.71–1.10	0.271	0.684	0.49–0.96	0.028	0.687	0.69–1.09	0.229
Neoantigen frequency	Per unit increase	1.144	1.01–1.29	0.030	1.125	1.00–1.26	0.047	1.185	1.03–1.36	0.016	1.127	0.99–1.28	0.067
IC50 ≤ 500	Per 100 unit increase	0.9848	0.93–1.04	0.536	0.991	0.95–1.03	0.657	0.973	0.92–1.03	0.367	0.984	0.94–1.03	0.496
IC50 < 50	Per 10 unit increase	0.991	0.96–1.02	0.542	0.993	0.97–1.01	0.517	0.986	0.95–1.02	0.415	0.988	0.96–1.01	0.332
IC50 < 34	Per unit increase	1.00	0.99–1.00	0.782	0.999	0.99–1.00	0.535	0.998	0.99–1.00	0.414	0.998	0.99–1.00	0.233
EL rank ≤ 2	Per 1000 unit increase	0.658	0.42–1.03	0.067	0.846	0.64–1.12	0.244	0.557	0.35–0.90	0.016	0.832	0.62–1.12	0.223
EL rank < 0.5	Per 100 unit increase	0.856	0.72–1.02	0.075	0.914	0.81–1.03	0.155	0.805	0.68–0.96	0.014	0.898	0.79–1.02	0.092
DAI ≥ 10	Per 10 unit increase	0.904	0.79–1.03	0.129	0.960	0.88–1.05	0.351	0.819	0.71–0.95	0.008	0.925	0.84–1.02	0.131
Dissimilarity ≥ 0.75	Per unit increase	0.964	0.90–1.04	0.325	0.970	0.92–1.02	0.266	0.950	0.87–1.03	0.229	0.963	0.91–1.02	0.212
IEDB ≥ 0.9	Per 100 unit increase	0.936	0.84–1.04	0.233	0.993	0.93–1.06	0.827	0.890	0.78–1.01	0.075	0.977	0.90–1.06	0.563
Neoantigen promiscuity	Per 100 unit increase	0.844	0.71–1.00	0.052	0.868	0.77–0.98	0.024	0.791	0.67–0.94	0.007	0.848	0.75–0.96	0.010
Driver genes	Per unit increase	1.01	0.99–1.03	0.414	1.015	1.00–1.03	0.077	1.008	0.98–1.03	0.520	1.015	1.00–1.03	0.117

^a^Mutlivariable analysis adjusted for gender, histology, stage, smoking status, EGFR and KRAS mutation status

### Higher neoantigen frequency is associated with shorter time to recurrence

As neoantigen load was significantly associated with a longer TTR, we hypothesised that patients with a higher proportion of mutations producing multiple neoantigens (referred to as neoantigen frequency) would also have improved outcomes. The median neoantigen frequency in this cohort was 6.4 (3.9–16.4).

However, higher neoantigen frequency was associated with shorter TTR and RFS in univariate analysis and, with shorter TTR in multivariate analysis (*p* = 0.016) (Fig. 3D and Table 2). When neoantigen load was included in the multivariate analysis, neoantigen frequency remained an independent predictor of shorter TTR (HR 1.168, *p* = 0.019). These results suggest that higher overall neoantigen quantity is associated with favourable prognosis, an elevated neoantigen frequency paradoxically indicates a higher risk of recurrence.

To further understand the mechanisms behind this finding, we evaluated whether neoantigen frequency correlated with any of the other neoantigen characteristics. The only significant correlation was between neoantigen frequency and the number of neoantigens with IC50 ≤ 500 (*r* = 0.303, *p* = 0.004) or IC50 < 50 (*r* = 0.303, *p* = 0.004). Notably, there was no correlation between neoantigen frequency and neoantigens with EL ≤ 2 (*r* =  − 0.030, *p* = 0.777) or EL < 0.5 (*r* =  − 0.028, *p* = 0.798), Furthermore, there was no correlation between neoantigen frequency and the proportions of passenger mutation-derived neoantigens (*r* = 0.031, *p* = 0.771).

### Neoantigens with high DAI and promiscuity are associated with improved clinical outcomes

Given the above association between neoantigen quantity and prognosis, we next evaluated how specific neoantigens properties influenced clinical outcome in early-stage NSCLC. We analysed each patient’s predicted neoantigens using qualitative immunogenicity metrics and correlated these with survival outcomes.

**Fig. 4:**
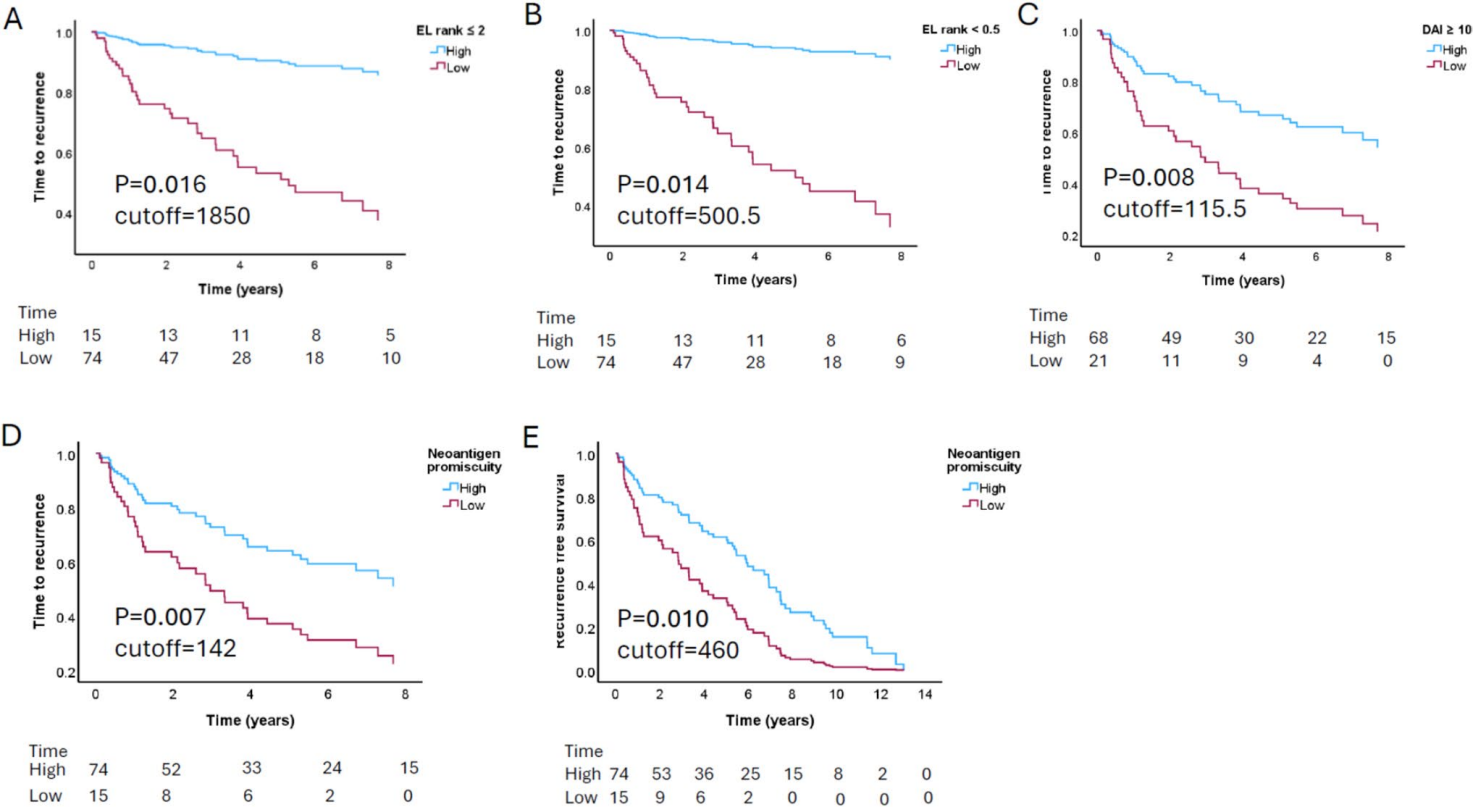
Neoantigens with EL rank ≤ 2, EL rank < 0.5, DAI ≥ 10 and neoantigen promiscuity correlate with improved prognosis in early-stage resected NSCLC (*n* = 89). Multivariate Cox regression analysis of TTR according to **a** EL rank ≤ 2, **b** EL rank < 0.5 and **c** DAI ≥ 10 and **d** TTR and **e** RFS according to neoantigen promiscuity. High and low groups are dichotomised by the optimal cutoff determined using Cutoff Finder. NSCLC, Non-small cell lung cancer; EL rank, Eluted ligand rank; DAI, Differential aggretopicity index; TTR, Time to recurrence; RFS, Recurrence-free survival

Neoantigens with EL ≤ 2 and EL < 0.5 were both associated with improved TTR (EL ≤ 2 *p* = 0.016, EL < 0.5 *p* = 0.014) (Fig. 4A, B Table 2). Similarly, patients who had more neoantigens with DAI ≥ 10, also had significantly longer TTR (*p* = 0.008) (Fig. 4C, Table 2). Neoantigen promiscuity was associated with both longer TTR (*p* = 0.007) and RFS (*p* = 0.010) (Fig. 4D, E Table 2). However, the number of HLA alleles per patient was not associated with prognosis (3–4 vs 5–6 HLA alleles, *p* = 0.418). In addition, we observed a trend towards better TTR in those with more neoantigens with IEDB ≥ 0.9, which reflects homology to infectious disease-derived epitopes (*p* = 0.075) (Table 2).

Other neoantigen properties, such as the number of neoantigens with either IC50 ≤ 500 or < 50, high dissimilarity to the non-mutated proteome and location in an oncogenic driver gene, were not associated with prognosis (Table 2). In addition, although the TESLA consortium reported IC50 < 34 as a predictor of immunogenicity [16], we found no correlation at this threshold with either TTR or RFS (Table 2).

### Prognostic value of neoantigens only observed in adenocarcinomas and not in squamous cell carcinomas

We next evaluated ADC and SCC as distinct subgroups as an exploratory analysis limited by the small patient numbers. Greater number of nsSNV, hvSNV and neoantigens were associated with improved clinical outcomes in the ADC but not SCC subgroup (Supp Tables 7 and 8). Higher number of neoantigens with a DAI ≥ 10 and neoantigen promiscuity were also associated with improved prognosis, and neoantigen frequency was associated with worse prognosis in the ADC subgroup (Supp Tables 7 and 8). These results require further validation in a larger patient cohort.

### Combining neoantigen property variables did not improve prognostic value compared to neoantigen load or individual variable

We assessed whether combining neoantigen properties with neoantigen load could improve the predictive ability for relapse risk. The median time to relapse in our cohort was 3 years. For this analysis, we included patients who had adequate follow-up of 3 years or more (*n* = 72).

Following logistic regression modelling and AUC analysis, we showed that the probability of being relapse-free at 3 years was predicted by neoantigen load (AUC = 0.760), lower neoantigen frequency (AUC = 0.723), DAI ≥ 10 (AUC = 0.744) and neoantigen promiscuity (AUC = 0.740) individually (Supp Table 9). There was no significant difference between the AUC values for these factors, and each risk model had good prediction performance. Combining the different metrics did not improve the prognostic relevance beyond that of each metric alone (Supp Table 9 and Supp Fig. 4).

Probabilities are computed by the logistic regression analysis (described in Statistical Analysis) and are adjusted for gender, TNM stage and smoking status.

### Neoantigen load was not associated with TCR repertoire clonality or diversity

Elevated TCR clonality has been suggested to reflect clonal expansion of antigen-specific T cells [35]. We hypothesised that higher neoantigen load would be associated with increased TCR clonality and a more diverse TCR repertoire. Using publicly available TCR sequencing data for this dataset, we correlated TCR diversity and clonality with predicted neoantigen load. The TCR diversity and clonality measures for each patient are shown in Fig. 2. There was no significant correlation between TCR diversity or clonality and neoantigen load (Supp Fig. 5A, B).

## Discussion

This study has demonstrated that nsSNV, hvSNV and neoantigen load are all strongly correlated and associated with favourable clinical outcomes in resected early-stage NSCLC. In contrast, neoantigen frequency, the number of neoantigens per mutation, was associated with poorer prognosis. In addition, high DAI and neoantigen promiscuity also predicted favourable clinical outcomes, supporting the importance of these neoantigen properties in mediating anti-tumour immunity.

A key finding from this study is that in early-stage lung cancer, the tumour’s nsSNV load, hvSNV load and neoantigen load were all positive prognostic factors. It is considered likely that during the early stages of tumour development, cytotoxic CD8 T cells eliminate immunogenic cancer cells [36]. Our results support this notion and suggest a role for neoantigen-specific immunosurveillance after resection. Although nsSNV load does not take into account the immunogenicity of the somatic mutations, it is strongly correlated with neoantigen load and is also associated with a lower risk of cancer relapse.

To ensure the comprehensiveness of our neoantigen prediction, we used both IC50 and EL rank. Neoantigen load was a predictor of clinical outcomes, consistent with prior early-stage NSCLC studies [8, 9]. Our results were mainly driven by neoantigens predicted by EL rank. EL rank integrates peptide-HLA affinity and peptide elution data, the latter accounts for antigen processing and presentation. The data are consistent with other studies showing the EL rank is superior to IC50 for neoantigen prediction [37, 38].

This study assessed several metrics of neoantigen quality to identify key features impacting patient outcome. DAI and neoantigen promiscuity were associated with delayed time to cancer relapse, whilst dissimilarity to self-antigens, similarity to known immunogenic antigens and derivation from driver oncogenes was not. DAI is a broad indicator of peptide dissimilarity from self and is, therefore, a likely indication of elevated immunogenicity. The previous studies including pan cancer, and in separate advanced NSCLC and melanoma cohorts [11, 13], have found also that high DAI correlated with improved patient survival.

The effect of neoantigen promiscuity on clinical outcomes has not been well studied. In our study, on average, 19.2% of neoantigens showed promiscuity for HLA binding. Similar, albeit slightly lower levels of neoantigen promiscuity were seen in patients with oesophagogastric adenocarcinoma [39], but the clinical relevance of this was not assessed in this study. Neoantigens that bind to multiple HLA alleles could theoretically increase the probability of successful neoantigen presentation to T cells, and perhaps recognition by different T-cell clones. The capacity to bind multiple alleles may also limit immune evasion by HLA loss of heterozygosity. Furthermore, our results show that the capacity of individual neoantigens to bind multiple alleles within a single patient was not a result of expression of alleles with similar peptide binding affinities, and 86% of all promiscuous neoantigens were predicted to bind to alleles from different genes or allele groups.

Unexpectedly, neoantigen frequency was a predictor for shorter TTR. Despite the apparent contradiction in outcomes associated with neoantigen load and neoantigen frequency, similar findings have been observed in ovarian cancer [40]. Matsushita and colleagues demonstrated that tumours enriched for mutations with a low neoantigen frequency expressed gene signatures consistent with a robust CD8-mediated immune response [40]. Whilst the mechanisms behind this counterintuitive finding remain elusive, possible explanations can be advanced. We show in our analysis that neoantigen frequency is positively correlated with the number of neoantigens predicted by IC50 but not EL rank. IC50 prediction is based on peptide-MHC binding affinity alone whereas EL rank is trained on mass spectrometry peptidome data and, therefore, also takes into account other steps in peptide antigen presentation, such as antigen processing and the stability of the peptide-MHC complex [41]. Studies examining evolutionary immune escape mechanisms in early-stage lung cancer and other cancers have demonstrated evidence for historical immune editing through disruption of the antigen presentation pathway, including downregulation of neoantigen expression, HLA loss of heterozygosity and epigenetic mechanisms such as promoter hypermethylation of neoantigen associated genes [42–44]. These immune evasion mechanisms have been associated with poor disease-free survival in early-stage lung cancer [42]. In our study, the lack of RNA sequencing data precludes further analysis of these immune escape pathways. However, it is possible that through selection pressure exerted by the immune microenvironment, genes associated with increased neoantigen frequency are affected by one or more of these pathways, resulting in neoantigens that are no longer immunogenic. Future studies with RNA sequencing data are warranted to investigate this hypothesis.

Another possible mechanism for the inverse association between neoantigen frequency and prognosis may be a mismatch between neoantigens that cross prime T cells to those directly presented on tumour cells. In viral infections, the hierarchy of cross- and directly-presented antigens can be quite distinct, with some immunodominant antigens presented better by direct presentation and some better by cross presentation [45]. This may also occur in the setting of cancer and high neoantigen frequency where uneven direct and cross presentation of variant peptides could compromise T-cell responses. Here, one could envisage that the efficacy of a T-cell primed to a specific peptide variant could be compromised by low expression on target cells, perhaps via competition for MHC binding with variant peptides derived from the high neoantigen frequency mutation. Importantly, appropriately targeted vaccines could bypass this constraint. Whatever the mechanism, these findings highlight unexpected nuances in the conversation between the immune response and the cancer genome.

In our study, neoantigen load was not associated with tumour TCR diversity or clonality, contrary to our hypothesis that high neoantigen load in tumours might promote expansion of tumour-specific T cells in the tumour microenvironment. Studies that examined this correlation have shown contradictory results. For example, studies in various cancer types, including lung, have also shown that T-cell clonality was independent of neoantigen load [46, 47] or tumour mutation burden [48, 49]. On contrary, mesothelioma and other lung cancer studies have shown a positive correlation between neoantigen load and TCR clonality [50, 51]. Notably, Reuben et al. showed in a cohort of 215 early-stage NSCLC patients that there was a weak positive correlation between TMB and TCR clonality (*r* = 0.19) [51]. Our study was conducted using a subset of this cohort (those with paired tumour and blood sequencing data) and found discrepant results. This may be related to a smaller number of patients (*n* = 89) resulting in a lack of statistical power. Another reason for the lack of correlation between neoantigen load and TCR diversity or clonality may be the low frequency of naturally occurring neoantigen-specific T cells in the tumour relative to bystander T cells that are responding to pathogens or other pro-inflammatory agents [52]. Therefore, clonal expansion of neoantigen-specific T cells may not be enough to significantly shift the overall TCR repertoire of the tumour. Moreover, our analysis of diversity and clonality do not take into account other TCR repertoire metrics that may be associated with antigen-specific T-cell responses, such as clonal relatedness, where TCRs with similar sequences and antigen specificity correlated with increased neoantigen load [47, 53].

The prognosis of early-stage NSCLC, even after surgical resection, is relatively poor, with up to 55% of all surgical patients developing recurrence within 5 years of diagnosis [1]. Therefore, a subset of these patients would derive benefit from additional therapies. Vaccines targeting neoantigens are promising strategies, and several clinical trials have demonstrated that neoantigen vaccines can induce T-cell responses in various cancer types [54, 55]. More recently, neoantigen vaccines were shown to significantly reduce the risk of cancer relapse in early-stage melanoma in combination with ICI, emphasising the considerable potential of neoantigens as targets in immunotherapy [56]. As well, adoptive transfer of autologous T-cell products containing high fractions of neoantigen-specific T cells has generated tumour regression in a range of cancer types [57, 58]. For these therapeutic approaches to be successful, it is critical immunoprotective antigens can be readily identified. Integrating neoantigen properties that enrich for immunogenicity has the potential to improve neoantigen prioritisation for the development of personalised immunotherapies. Based on our results, prioritising neoantigens with high DAI and the ability to bind multiple HLA alleles may improve therapeutic efficacy.

Our study has several limitations that highlight areas for future research. Firstly, our analysis was performed using the best studied mutation type nsSNVs; however, other genetic aberrations may produce comparable or even more immunogenic neoantigens, such as gene fusions [59], splice variants [60] and chromosomal rearrangements [61]. However, these complex neoantigens are difficult to identify comprehensively and accurately using the currently available bioinformatic tools, despite the evolving interest in their role as contributors to anti-cancer immunity and, therefore, were excluded from this study. Secondly, we did not examine other characteristics that have been shown to potentially modulate neoantigen immunogenicity including peptide-MHC binding stability [16], and gene expression—a key determinant of immunogenicity [16], the latter due to lack of RNA sequencing data [62, 63]. Importantly, there were incomplete clinical data such as patient age, performance status and adjuvant treatment, which may confound clinical outcomes. Lastly, we used a single-centre dataset in a limited Caucasian population which restricts the generalisability of our findings. Future work in a larger multi-centre cohort considering other clinical characteristics and treatments, with the inclusion gene expression analysis, is needed to validate our findings presented here.

This study focuses on early-stage surgically resected NSCLC patients, an extremely important group of lung cancer patients where a better understanding of prognostic biomarkers could lead to a higher cure rate. To the best of our knowledge, this study is the first to evaluate and successfully identify neoantigen properties associated with cancer outcomes in this patient cohort. We anticipate improved neoantigen prioritisation for the development of personalised immunotherapies, where integration of mutant to wildtype peptide-MHC binding affinity and HLA allele binding data may improve the precision of neoantigen selection. Further research will be needed to fully profile the response to personalised immunotherapy targeting neoantigens with these properties.

This study provides evidence of neoantigen-related immune protection in early-stage NSCLC. Mutation load and predicted neoantigen load were both useful indicators of prognosis and provided effective stratification variables in prognosticating disease outcome. Additionally, our results highlight the importance of not only neoantigen quantity, but also specific neoantigen properties for the prognostication of early-stage NSCLC and provide important insight for the rational selection of neoantigens for therapeutic targeting.

## Supplementary Information

Below is the link to the electronic supplementary material.

Supplementary file1 (DOCX 1654 kb)

## Data Availability

The datasets generated and/or analysed during the current study are available from the corresponding author on reasonable request.
