# Supplementary material for: Neoantigen load as a predictor of relapse in early-stage NSCLC: features that agonise and antagonise prognosis
Source: Cancer Immunol Immunother. 2025 Aug 6;74(9):285. doi: 10.1007/s00262-025-04131-y (PMC12328875; doi:10.1007/s00262-025-04131-y)
Supplement: Supplementary file 1 — Supplementary file1 (DOCX 1654 kb) [file 262_2025_4131_MOESM1_ESM.docx]

**Supp Fig. 1** Strong positive linear correlation between mutational and neoantigen load in early-stage resected NSCLC (n=89). a) nsSNV load and hvSNV load b) nsSNV load and predicted neoantigen load and c) hvSNV load and predicted neoantigen load. Spearman’s correlation

NSCLC: non-small cell lung cancer; nsSNV: non-synonymous single nucleotide variant; hvSNV: high value non-synonymous single nucleotide variant


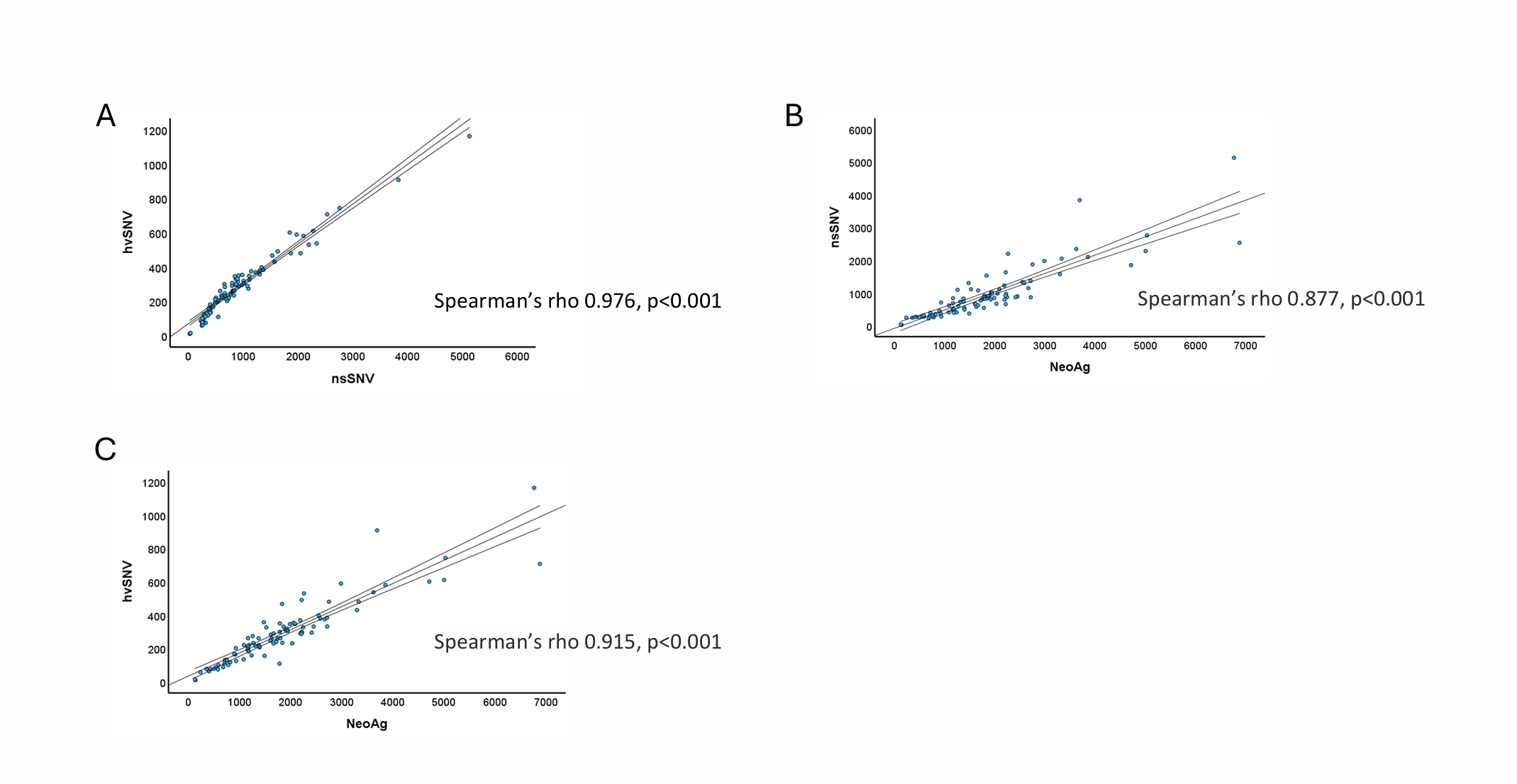


**Supp Fig. 2** Percentages of all predicted neoantigens and absolute numbers of predicted neoantigens based on a) EL rank b) IC50 c) DAI ≥ 10 d) Dissimilarity score ≥ 0.75 e) IEDB score ≥ 0.9 f) neoantigen promiscuity g) oncogene derived

EL rank: eluted ligand rank; DAI: differential aggretopicity index; IEDB: Immune Epitope Database


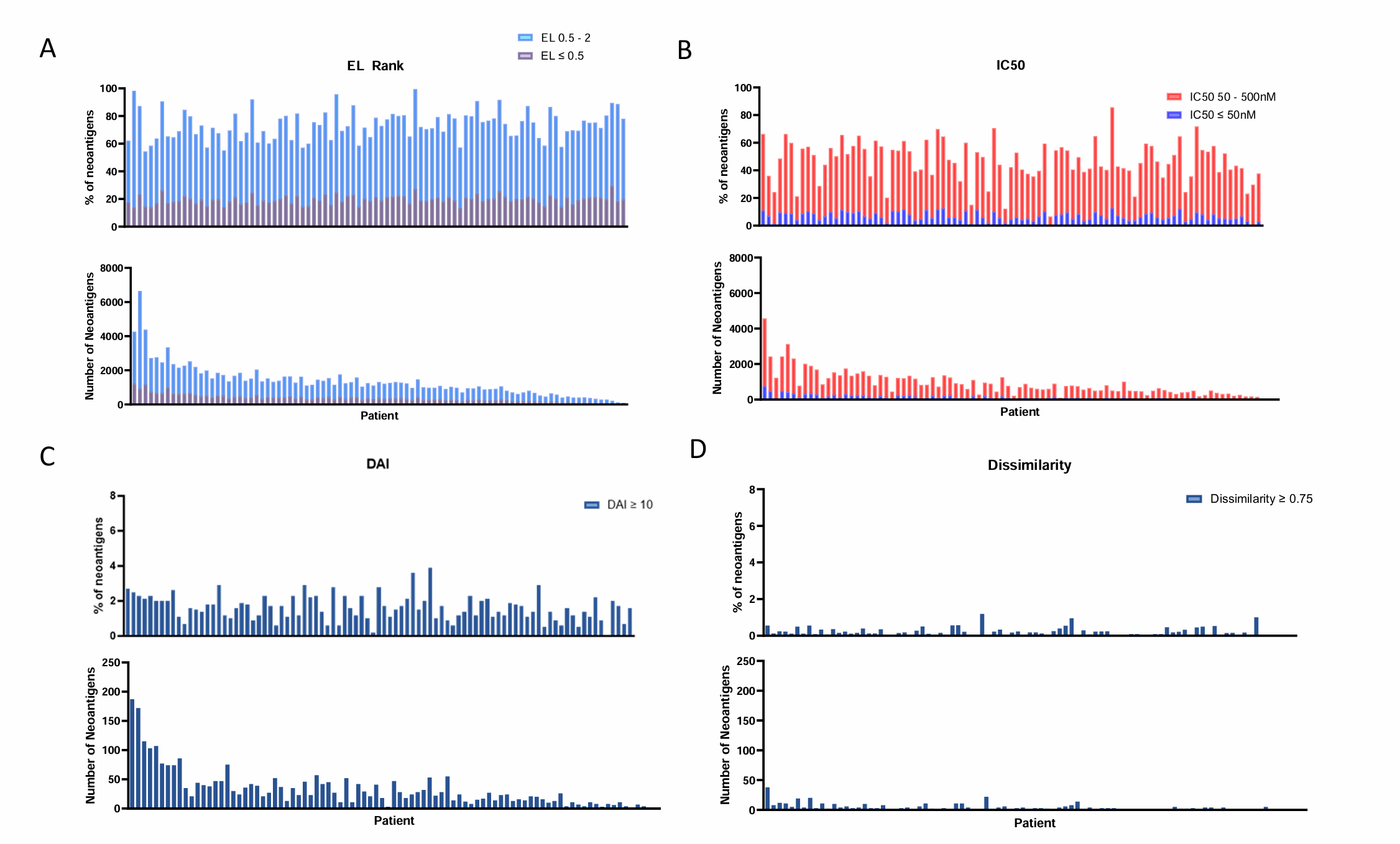


*
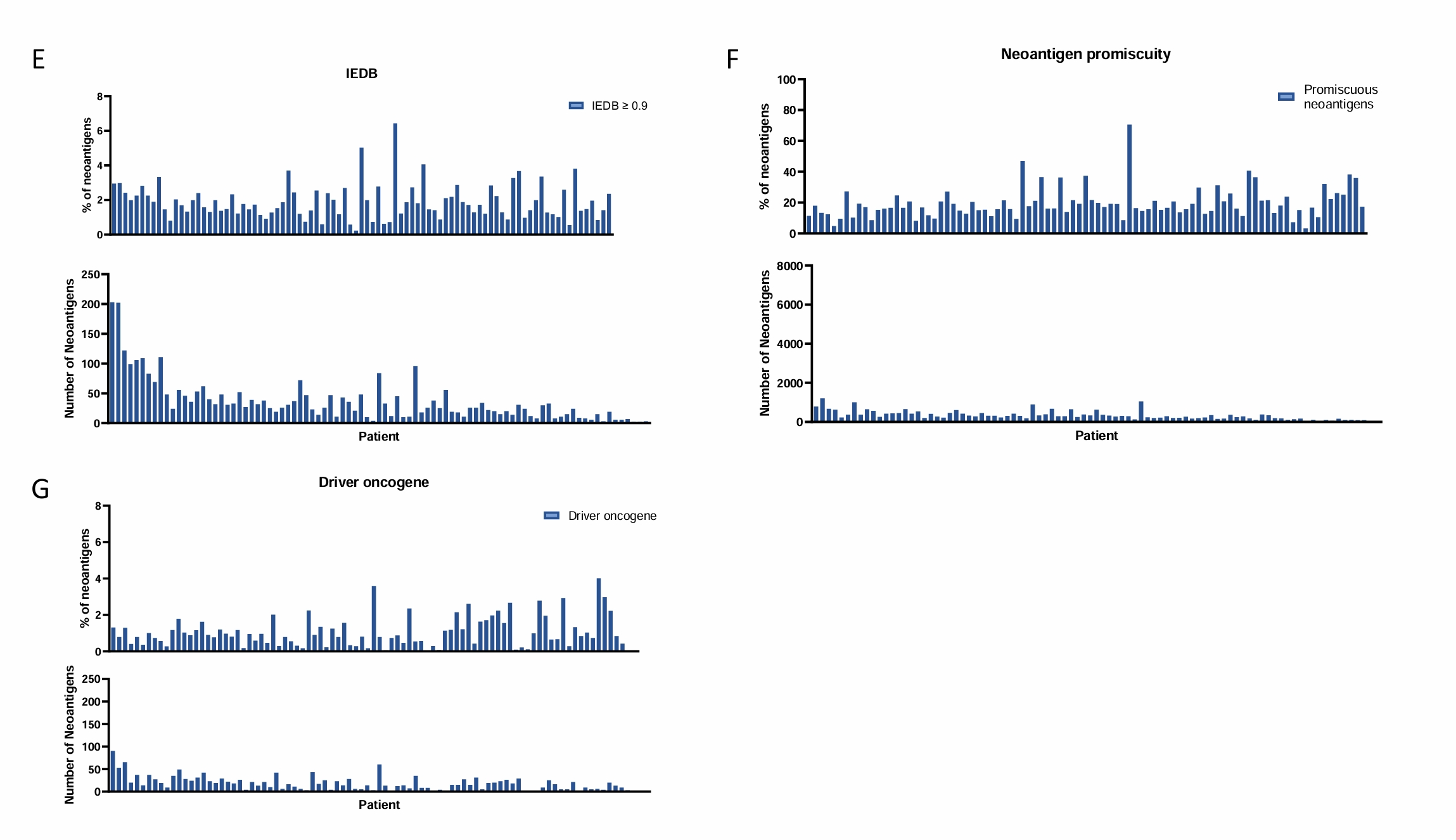
*

**Supp Fig. 3** Number of promiscuous neoantigens predicted to bind to each HLA gene

HLA: human leukocyte antigen

*
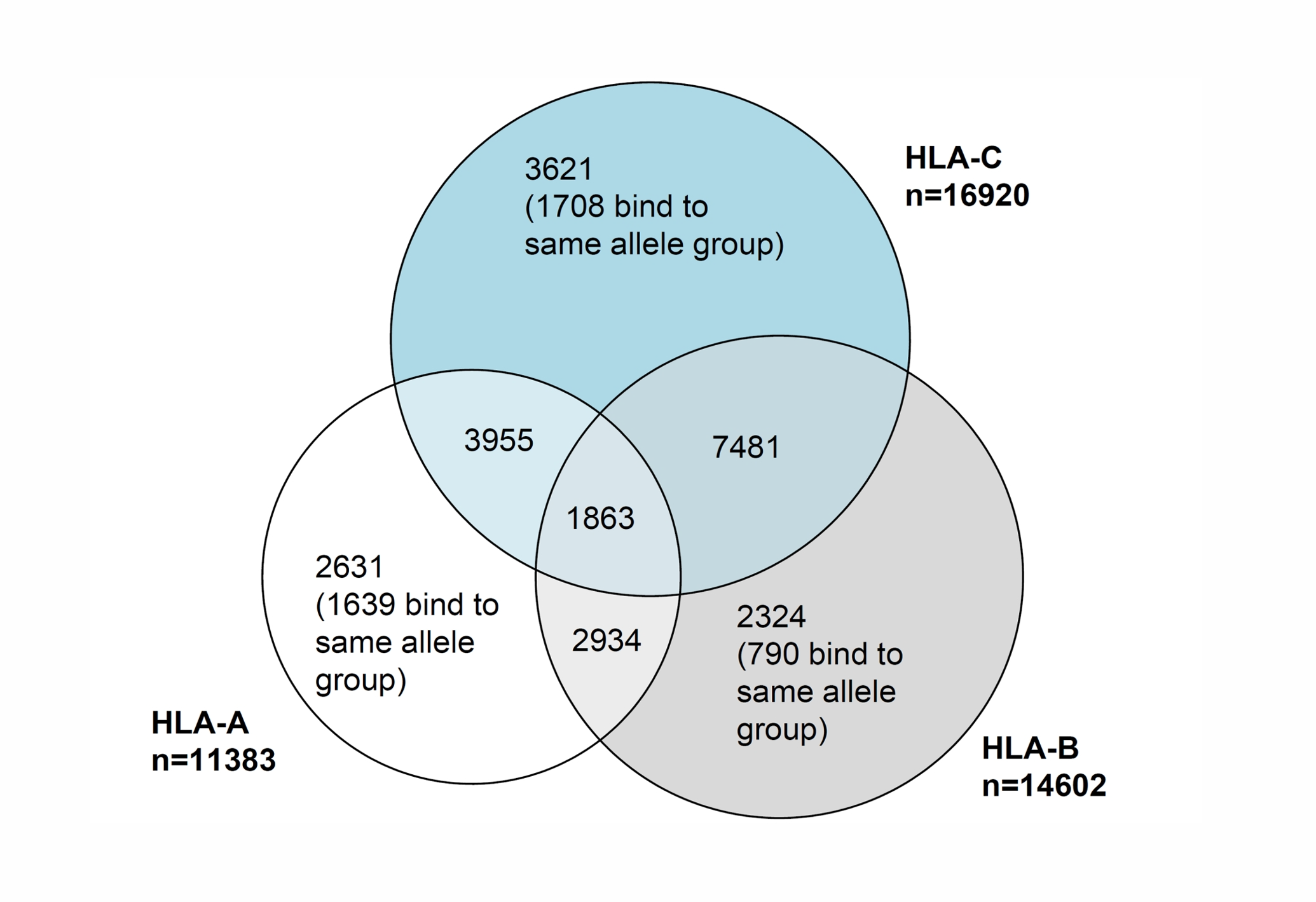
*

**Supp Fig. 4** AUC analysis of the probability of an individual being recurrence free at 3 years of predicted neoantigen load alone and in combination with neoantigen frequency, DAI ≥ 10 and neoantigen promiscuity

AUC: area under the curve, DAI: differential aggretopicity index


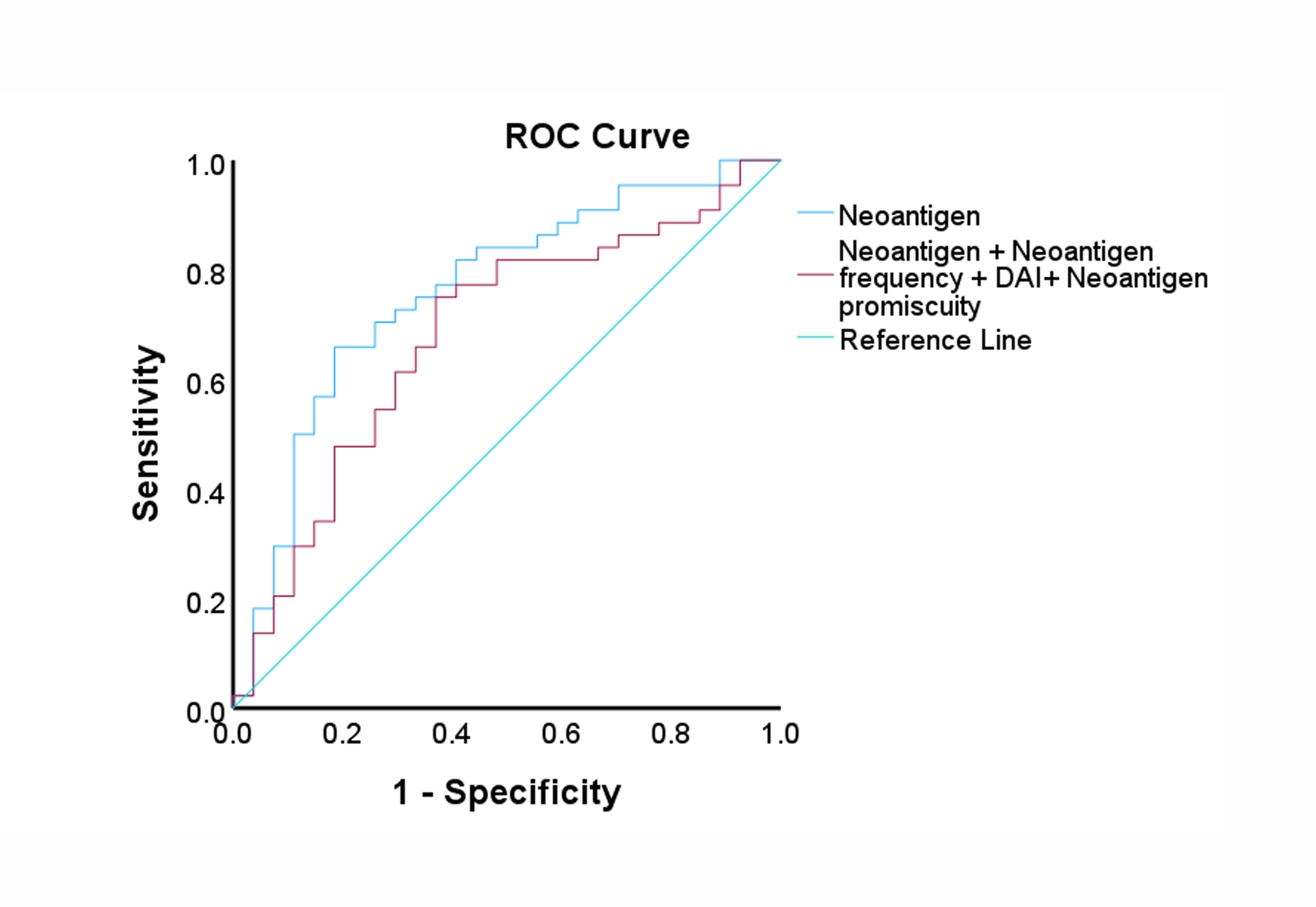


**Supp Fig. 5** No correlation between predicted neoantigen load and A) TCR diversity or B) clonality in early stage resected NSCLC (n=89)

TCR: T cell receptor; NSCLC: non-small cell lung cancer


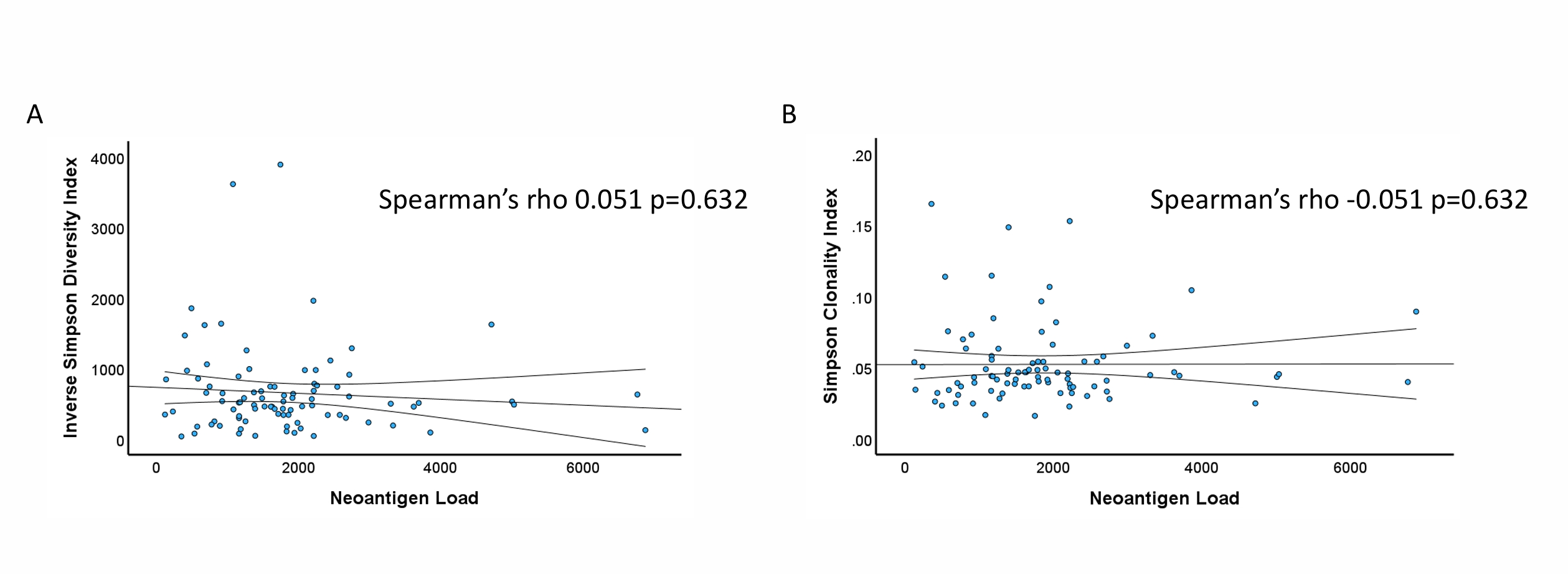


**Supp Table 1. Number of hvSNV and nsSNV-derived neoantigens predicted to originate from known NSCLC oncogenes**

| **Oncogene** | **hvSNV** | **Neoantigens** |
| --- | --- | --- |
|  |  |  |
| CSMD3 | 31 | 288 |
| TP53 | 42 | 251 |
| KEAP1 | 11 | 72 |
| KRAS | 19 | 67 |
| GRIN2A | 7 | 65 |
| BIRC6 | 13 | 64 |
| PTPRT | 7 | 59 |
| NOTCH1 | 21 | 53 |
| PTPRD | 9 | 49 |
| EPHA3 | 7 | 40 |
| EGFR | 9 | 36 |
| MAP2K1 | 3 | 34 |
| BRAF | 3 | 30 |
| SMARCA4 | 5 | 30 |
| RET | 4 | 29 |
| N4BP2 | 4 | 27 |
| NFE2L2 | 4 | 26 |
| PTPN13 | 3 | 26 |
| STK11 | 4 | 25 |
| ARAF | 2 | 21 |
| DDR2 | 5 | 21 |
| DROSHA | 1 | 20 |
| ROS1 | 3 | 20 |
| TPR | 2 | 18 |
| RFWD3 | 4 | 17 |
| KDR | 5 | 16 |
| CUL3 | 2 | 14 |
| ERBB4 | 3 | 13 |
| HIP1 | 3 | 12 |
| PIK3CB | 4 | 12 |
| RAD21 | 2 | 12 |
| EML4 | 1 | 10 |
| RBM10 | 4 | 10 |
| TP63 | 2 | 10 |
| CD74 | 1 | 9 |
| FGFR2 | 1 | 8 |
| MYCL | 1 | 8 |
| NRG1 | 4 | 6 |
| TPM3 | 1 | 6 |
| SIRPA | 1 | 4 |
| SLC34A2 | 1 | 3 |
| MB21D2 | 1 | 2 |
| ZNF479 | 1 | 2 |
| USP44 | 1 | 1 |
| AKT1 | 0 | 0 |
| BAP1 | 0 | 0 |
| CCDC6 | 0 | 0 |
| CPEB3 | 0 | 0 |
| EED | 0 | 0 |
| ERBB2 | 0 | 0 |
| EZR | 0 | 0 |
| GPC5 | 0 | 0 |
| HIF1A | 0 | 0 |
| KIF5B | 0 | 0 |
| LEPROTL1 | 0 | 0 |
| LRIG3 | 0 | 0 |
| MALAT1 | 0 | 0 |
| MAP2K2 | 0 | 0 |
| NKX2-1 | 0 | 0 |
| RB1 | 0 | 0 |
| SDC4 | 0 | 0 |
| SOX2 | 0 | 0 |
| STRN | 0 | 0 |
| TFG | 0 | 0 |

**Supp Table 2. Neoantigen landscape of adenocarcinomas and squamous cell carcinomas**

| **Variable** | **ADC** | **SCC** | **p-value** |
| --- | --- | --- | --- |
| nsSNV load [median (range)] | 797 (25 – 5126) | 816 (255 – 1849) | 0.465 |
| hvSNV load [median (range)] | 234 (10 – 1163) | 291 (125 – 601) | 0.181 |
| Neoantigen load [median (range)] | 1492 (127 – 6882) | 1735 (931 – 4718) | 0.283 |
| IC50 ≤ 500nM (%) | 48.1 | 47.2 | 0.656 |
| IC50 < 50nM (%) | 7.4 | 7.0 | 0.500 |
| EL rank ≤ 2 (%) | 73.4 | 71.6 | 0.268 |
| EL rank < 0.5 (%) | 18.8 | 18.9 | 0.267 |
| DAI ≥ 10 (%) | 1.9 | 1.4 | 0.653 |
| Dissimilarity ≥ 0.75 (%) | 0.3 | 0.2 | 0.511 |
| IEDB ≥ 0.9 (%) | 2.2 | 1.6 | 0.582 |
| Neoantigen promiscuity (%) | 17.7 | 18.1 | 0.254 |
| Oncogenic driver genes (%) | 1.1 | 0.8 | 0.140 |

**Supp Table 3. Neoantigen landscape and cancer stage**

| **Variable** | **I** | **II** | **III** | **p-value** |
| --- | --- | --- | --- | --- |
| nsSNV load [median (range)] | 769 (25 – 5126) | 816 (245 – 2757) | 660 (40 – 3831) | 0.710 |
| hvSNV load [median (range)] | 239 (10 – 1163) | 286 (74 – 743) | 271 (15 – 908) | 0.646 |
| Neoantigen load [median (range)] | 1527 (142 – 6770) | 1771 (358 – 5035) | 1781 (127 – 6882) | 0.512 |
| IC50 ≤ 500nM (%) | 45.3 | 50.6 | 44.1 | 0.397 |
| IC50 < 50nM (%) | 6.3 | 7.4 | 6.0 | 0.399 |
| EL rank ≤ 2 (%) | 73.2 | 71.5 | 75.7 | 0.852 |
| EL rank < 0.5 (%) | 19.2 | 19.6 | 19.7 | 0.645 |
| DAI ≥ 10 (%) | 1.5 | 1.8 | 1.4 | 0.188 |
| Dissimilarity ≥ 0.75 (%) | 0.1 | 0.2 | 0.1 | 0.588 |
| IEDB ≥ 0.9 (%) | 1.5 | 2.0 | 1.7 | 0.196 |
| Neoantigen promiscuity (%) | 16.6 | 16.6 | 18.5 | 0.845 |
| Oncogenic driver genes (%) | 0.8 | 0.8 | 0.9 | 0.789 |

**Supp Table 4. Neoantigen landscape and smoking**

| **Variable** | **Smoker** | **Never smoker** | **p-value** |
| --- | --- | --- | --- |
| nsSNV load [median (range)] | 830 (40 – 5126) | 256 (25 – 551) | <0.001 |
| hvSNV load [median (range)] | 287 (15 – 1163) | 83 (10 – 207) | <0.001 |
| Neoantigen load [median (range)] | 1792 (127 – 6882) | 591 (142 – 1394) | <0.001 |
| IC50 ≤ 500nM (%) | 46.4 | 53.5 | 0.523 |
| IC50 < 50nM (%) | 6.3 | 6.6 | 0.803 |
| EL rank ≤ 2 (%) | 73.5 | 70.9 | 0.680 |
| EL rank < 0.5 (%) | 19.7 | 19.6 | 0.935 |
| DAI ≥ 10 (%) | 1.6 | 1.5 | 0.692 |
| Dissimilarity ≥ 0.75 (%) | 0.1 | 0.1 | 0.321 |
| IEDB ≥ 0.9 (%) | 1.8 | 1.4 | 0.537 |
| Neoantigen promiscuity (%) | 17.0 | 20.6 | 0.629 |
| Oncogenic driver genes (%) | 0.9 | 0.5 | 0.456 |

**Supp Table 5. Neoantigen landscape and EGFR mutation**

| **Variable** | **EGFR mutant** | **EGFR wildtype** | **p-value** |
| --- | --- | --- | --- |
| nsSNV load [median (range)] | 310 (225 – 5126) | 829 (25 – 3831) | 0.002 |
| hvSNV load [median (range)] | 111 (63 – 1163) | 285 (15 – 908) | 0.001 |
| Neoantigen load [median (range)] | 707 (406 – 6770) | 1789 (127 – 6882) | 0.002 |
| IC50 ≤ 500nM (%) | 51.1 | 48.1 | 0.763 |
| IC50 < 50nM (%) | 6.9 | 6.2 | 0.963 |
| EL rank ≤ 2 (%) | 71.4 | 73.3 | 0.498 |
| EL rank < 0.5 (%) | 20.2 | 19.7 | 0.357 |
| DAI ≥ 10 (%) | 1.2 | 1.7 | 0.069 |
| Dissimilarity ≥ 0.75 (%) | 0.1 | 0.1 | 0.374 |
| IEDB ≥ 0.9 (%) | 1.5 | 1.8 | 0.732 |
| Neoantigen promiscuity (%) | 21.5 | 16.7 | 0.372 |
| Oncogenic driver genes (%) | 1.9 | 0.8 | 0.038 |

**Supp Table 6. Neoantigen landscape and KRAS mutation**

| **Variable** | **KRAS mutant** | **KRAS wildtype** | **p-value** |
| --- | --- | --- | --- |
| nsSNV load [median (range)] | 800 (247 – 2277) | 800 (25 – 5126) | 0.844 |
| hvSNV load [median (range)] | 234 (58 – 611) | 268 (15 – 1163) | 0.556 |
| Neoantigen load [median (range)] | 1670 (238 – 5008) | 1694 (127 – 6882) | 0.837 |
| IC50 ≤ 500nM (%) | 53.5 | 45.9 | 0.412 |
| IC50 < 50nM (%) | 6.2 | 6.5 | 0.644 |
| EL rank ≤ 2 (%) | 72.1 | 73.3 | 0.762 |
| EL rank < 0.5 (%) | 19.7 | 19.8 | 0.893 |
| DAI ≥ 10 (%) | 2.0 | 1.5 | 0.043 |
| Dissimilarity ≥ 0.75 (%) | 0.1 | 0.1 | 0.351 |
| IEDB ≥ 0.9 (%) | 1.9 | 1.7 | 0.942 |
| Neoantigen promiscuity (%) | 18.0 | 16.8 | 0.846 |
| Oncogenic driver genes (%) | 1.6 | 1.4 | 0.521 |

**Supp Table 7. Multivariate analysis of TTR and RFS predictors in adenocarcinomas (n=59)**

| **Variables** |  | **Univariate** | | | | | | **Multivariate^a^** | | | | | |
| --- | --- | --- | --- | --- | --- | --- | --- | --- | --- | --- | --- | --- | --- |
|  |  | **TTR** | | | **RFS** | | | **TTR** | | | **RFS** | | |
|  |  | **HR** | **95% CI** | **P** | **HR** | **95% CI** | **p** | **HR** | **95% CI** | **p** | **HR** | **95% CI** | **p** |
| Gender | F vs M | 0.630 | 0.30 – 1.33 | 0.224 | 0.857 | 0.48 – 1.52 | 0.598 |  |  |  |  |  |  |
| Stage | I |  |  |  |  |  |  |  |  |  |  |  |  |
|  | II | 3.129 | 1.32 – 7.43 | 0.010 | 1.450 | 0.70 – 2.97 | 0.310 |  |  |  |  |  |  |
|  | III | 3.649 | 1.50 – 8.85 | 0.004 | 1.505 | 0.72 – 3.16 | 0.280 |  |  |  |  |  |  |
| EGFR | Mutant vs WT | 0.420 | 0.10 – 1.77 | 0.237 | 1.161 | 0.51 – 2.62 | 0.719 |  |  |  |  |  |  |
| KRAS | Mutant vs WT | 1.612 | 0.78 – 3.32 | 0.196 | 1.062 | 0.59 – 1.92 | 0.843 |  |  |  |  |  |  |
| Smoking | Ever vs Never | 0.930 | 0.20 – 3.09 | 0.905 | 0.511 | 0.21 – 1.26 | 0.142 |  |  |  |  |  |  |
| nsSNV load | Per 100 unit increase | 0.933 | 0.88 – 0.99 | 0.024 | 0.968 | 0.94 – 1.00 | 0.062 | 0.916 | 0.86 – 0.98 | 0.008 | 0.967 | 0.93 – 1.00 | 0.063 |
| hvSNV load | Per 100 unit increase | 0.791 | 0.64 – 0.98 | 0.032 | 0.874 | 0.76 – 1.01 | 0.063 | 0.733 | 0.58 – 0.93 | 0.009 | 0.872 | 0.75 – 1.01 | 0.068 |
| Neoantigen load | Per 1000 unit increase | 0.755 | 0.5 – 1.04 | 0.089 | 0.830 | 0.66 – 1.05 | 0.117 | 0.669 | 0.72 – 0.96 | 0.028 | 0.827 | 0.64 – 1.06 | 0.138 |
| Neoantigen frequency | Per unit increase | 1.121 | 0.98 – 1.28 | 0.090 | 1.093 | 0.96 – 1.25 | 0.182 | 1.173 | 1.00 – 1.37 | 0.043 | 1.101 | 0.95 – 1.27 | 0.185 |
| IC50 ≤ 500 | Per 100 unit increase | 0.974 | 0.92 – 1.03 | 0.383 | 0.972 | 0.0.93 – 1.02 | 0.227 | 0.952 | 0.89 – 1.02 | 0.177 | 0.965 | 0.92 – 1.02 | 0.195 |
| IC50 < 50 | Per 10 unit increase | 0.983 | 0.95 – 1.01 | 0.286 | 0.980 | 0.96 – 1.00 | 0.124 | 0.971 | 0.93 – 1.01 | 0.138 | 0.975 | 0.95 – 1.00 | 0.089 |
| IC50 < 34 | Per unit increase | 0.998 | 0.99 – 1.00 | 0.414 | 0.997 | 0.99 – 1.00 | 0.109 | 0.995 | 0.99 – 1.00 | 0.115 | 0.995 | 0.99 – 1.00 | 0.043 |
| EL rank ≤ 2 | Per 1000 unit increase | 0.668 | 0.42 – 1.05 | 0.080 | 0.810 | 0.61 – 1.08 | 0.156 | 0.562 | 0.34 – 0.93 | 0.026 | 0.817 | 0.60 – 1.11 | 0.198 |
| EL rank < 0.5 | Per 100 unit increase | 0.857 | 0.72 – 1.02 | 0.083 | 0.894 | 0.79 – 1.02 | 0.088 | 0.807 | 0.60 – 0.97 | 0.021 | 0.888 | 0.78 – 1.01 | 0.082 |
| DAI ≥ 10 | Per 10 unit increase | 0.889 | 0.77 – 1.02 | 0.094 | 0.929 | 0.84 – 1.02 | 0.131 | 0.801 | 0.68 – 0.94 | 0.007 | 0.907 | 0.81 – 1.01 | 0.085 |
| Dissimilarity ≥ 0.75 | Per unit increase | 0.950 | 0.88 – 1.03 | 0.206 | 0.952 | 0.90 – 1.01 | 0.117 | 0.950 | 0.87 – 1.04 | 0.256 | 0.959 | 0.90 – 1.02 | 0.201 |
| IEDB ≥ 0.9 | Per 10 unit increase | 0.927 | 0.83 – 1.04 | 0.197 | 0.961 | 0.89 – 1.04 | 0.317 | 0.885 | 0.77 – 1.01 | 0.081 | 0.956 | 0.88 – 1.04 | 0.315 |
| Neoantigen promiscuity | Per 100 unit increase | 0.820 | 0.68 – 0.98 | 0.032 | 0.836 | 0.72 – 0.95 | 0.008 | 0.782 | 0.65 – 0.94 | 0.010 | 0.832 | 0.73 – 0.95 | 0.008 |
| Driver Genes | Per unit increase | 1.001 | 0.98 – 1.02 | 0.926 | 1.008 | 0.99 – 1.03 | 0.364 | 1.000 | 0.97 – 1.02 | 0.891 | 1.01 | 0.99 – 1.03 | 0.253 |

^a^Multivariable analysis adjusted for gender, stage, smoking, EGFR and KRAS mutation status

**Supp Table 8. Multivariate analysis of TTR and RFS predictors in squamous cell carcinomas (n=30)**

| **Variables** |  | **Univariate** | | | | | | **Multivariate^a^** | | | | | |
| --- | --- | --- | --- | --- | --- | --- | --- | --- | --- | --- | --- | --- | --- |
|  |  | **TTR** | | | **RFS** | | | **TTR** | | | **RFS** | | |
|  |  | **HR** | **95% CI** | **P** | **HR** | **95% CI** | **p** | **HR** | **95% CI** | **p** | **HR** | **95% CI** | **p** |
| Gender | F vs M | 0.162 | 0.02 – 1.30 | 0.087 | 0.829 | 0.30 – 2.26 | 0.714 |  |  |  |  |  |  |
| Stage | I |  |  |  |  |  |  |  |  |  |  |  |  |
|  | II | 1.176 | 0.26 – 5.27 | 0.833 | 1.538 | 0.53 – 4.48 | 0.430 |  |  |  |  |  |  |
|  | III | 6.502 | 0.87 – 48.82 | 0.069 | 3.352 | 0.56 – 19.92 | 0.183 |  |  |  |  |  |  |
| nsSNV load | Per 100 unit increase | 0.878 | 0.69 – 1.12 | 0.275 | 0.975 | 0.84 – 1.13 | 0.734 | 0.878 | 0.68 – 1.14 | 0.326 | 0.963 | 0.82 – 1.13 | 0.639 |
| hvSNV load | Per 100 unit increase | 0.598 | 0.25 – 1.4 | 0.241 | 0.859 | 0.49 – 1.51 | 0.598 | 0.534 | 0.20 – 1.43 | 0.213 | 0.773 | 0.41 – 1.45 | 0.423 |
| Neoantigen load | Per 1000 unit increase | 0.77 | 0.30 – 2.03 | 0.608 | 0.856 | 0.54 – 2.09 | 1.064 | 0.754 | 0.20 – 2.78 | 0.671 | 1.036 | 0.50 – 2.13 | 0.922 |
| Neoantigen frequency | Per unit increase | 1.487 | 0.78 – 2.82 | 0.225 | 1.520 | 0.87 – 2.67 | 0.144 | 1.564 | 0.78 – 3.12 | 0.205 | 1.699 | 0.93 – 3.08 | 0.082 |
| IC50 ≤ 500 | Per 100 unit increase | 0.996 | 0.90 – 1.10 | 0.944 | 1.015 | 0.93 – 1.10 | 0.726 | 1.011 | 0.88 – 1.16 | 0.87 | 1.014 | 0.93 – 1.11 | 0.754 |
| IC50 < 50 | Per 10 unit increase | 1.012 | 0.96 – 1.07 | 0.682 | 1.020 | 0.97 – 1.07 | 0.470 | 1.033 | 0.96 – 1.12 | 0.408 | 1.021 | 0.97 – 1.08 | 0.466 |
| IC50 < 34 | Per unit increase | 1.003 | 0.99 – 1.01 | 0.522 | 1.004 | 1.00 – 1.01 | 0.365 | 1.007 | 0.99 – 1.02 | 0.257 | 1.004 | 1.00 – 1.01 | 0.355 |
| EL rank ≤ 2 | Per 1000 unit increase | 0.547 | 0.11 – 2.71 | 0.460 | 0.919 | 0.29 – 2.91 | 0.886 | 0.435 | 0.06 – 3.26 | 0.418 | 0.786 | 0.22 – 2.83 | 0.713 |
| EL rank < 0.5 | Per 100 unit increase | 0.829 | 0.45 – 1.52 | 0.547 | 0.960 | 0.61 – 1.52 | 0.860 | 0.758 | 0.35 – 1.64 | 0.481 | 0.902 | 0.54 – 1.50 | 0.690 |
| DAI ≥ 10 | Per 10 unit increase | 0.886 | 0.59 – 1.33 | 0.557 | 1.009 | 0.77 – 1.32 | 0.949 | 0.862 | 0.44 – 1.67 | 0.660 | 0.993 | 0.74 – 1.33 | 0.964 |
| Dissimilarity ≥ 0.75 | Per unit increase | 1.008 | 0.78 – 1.30 | 0.950 | 0.949 | 0.78 – 1.15 | 0.602 | 0.950 | 0.66 – 1.36 | 0.781 | 0.948 | 0.77 – 1.17 | 0.616 |
| IEDB ≥ 0.9 | Per 10 unit increase | 0.898 | 0.65 – 1.24 | 0.519 | 1.076 | 0.87 – 1.34 | 0.504 | 0.974 | 0.64 – 1.48 | 0.900 | 1.077 | 0.81 – 1.42 | 0.605 |
| Neoantigen promiscuity | Per 100 unit increase | 1.077 | 0.64 – 1.82 | 0.781 | 0.986 | 0.66 – 1.47 | 0.944 | 0.882 | 0.48 – 1.60 | 0.680 | 0.906 | 0.58 – 1.41 | 0.662 |
| Driver Genes | Per unit increase | 1.031 | 0.98 – 1.09 | 0.243 | 1.030 | 0.98 – 1.08 | 0.214 | 1.081 | 1.00 – 1.17 | 0.062 | 1.036 | 0.98 – 1.09 | 0.189 |

^a^Multivariable analysis adjusted for gender, stage. All patients were smokers and EGFR and KRAS wildtype.

**Supp Table 9. AUC analysis of the probability of an individual being recurrence free at 3 years of predicted neoantigen load, DAI≥10 and neoantigen promiscuity individually and in combination**

| **Biomarker** | **AUC (95% CI)** |
| --- | --- |
| Neoantigen | 0.760 (0.642 – 0.877) |
| Neoantigen frequency | 0.723 (0.600 – 0.845) |
| DAI | 0.744 (0.622– 0.865) |
| Neoantigen promiscuity | 0.740 (0.618 – 0.863) |
| Neoantigen + Neoantigen frequency | 0.711 (0.582 – 0.840) |
| Neoantigen + DAI | 0.740 (0.617 – 0.863) |
| Neoantigen + Neoantigen promiscuity | 0.745 (0.624 - 0.866) |
| Neoantigen frequency + DAI | 0.691 (0.559 – 0.823) |
| Neoantigen frequency + Neoantigen promiscuity | 0.709 (0.581 – 0.836) |
| DAI + Neoantigen promiscuity | 0.682 (0.551 – 0.813) |
| Neoantigen + Neoantigen frequency + DAI | 0.688 (0.555 – 0.820) |
| Neoantigen + Neoantigen frequency + Neoantigen promiscuity | 0.709 (0.583 – 0.836) |
| Neoantigen + DAI + Neoantigen promiscuity | 0.645 (0.521 – 0.787) |
| Neoantigen frequency + DAI + Neoantigen promiscuity | 0.691 (0.560 – 0.823) |
| Neoantigen + Neoantigen frequency + DAI + Neoantigen promiscuity | 0.680 (0.550 – 0.810) |
